# Peripheral Mechanobiology of Touch—Studies on Vertebrate Cutaneous Sensory Corpuscles

**DOI:** 10.3390/ijms21176221

**Published:** 2020-08-27

**Authors:** Ramón Cobo, Jorge García-Piqueras, Yolanda García-Mesa, Jorge Feito, Olivia García-Suárez, Jose A Vega

**Affiliations:** 1 Departamento de Morfología y Biología Celular, Grupo SINPOS, Universidad de Oviedo, Avda. Julian Clavería. 6, 33006 Oviedo, Spain; ramoncobodiaz@gmail.com (R.C.); garciapiquerasjorge@gmail.com (J.G.-P.); yolandagm_navia@hotmail.com (Y.G.-M.); garciaolivia@uniovi.es (O.G.-S.); 2 Servicio de Anatomía Patológica, Hospital Clínico—Complejo Asistencial de Salamanca, 37007 Salamanca, Spain; jfeito@saludcastillayleon.es; 3 Departamento de Anatomía e Histología Humanas, Universidad de Salamanca, 37007 Salamanca, Spain; 4 Facultad de Ciencias de la Salud, Universidad Autónoma de Chile, Providencia, 6640022 Santiago de Chile, Chile

**Keywords:** skin, sensory corpuscles, low-threshold mechanoreceptors, mechanoproteins, acid-sensing ion channels, transient receptor potential channels, Piezo2

## Abstract

The vertebrate skin contains sensory corpuscles that are receptors for different qualities of mechanosensitivity like light brush, touch, pressure, stretch or vibration. These specialized sensory organs are linked anatomically and functionally to mechanosensory neurons, which function as low-threshold mechanoreceptors connected to peripheral skin through Aβ nerve fibers. Furthermore, low-threshold mechanoreceptors associated with Aδ and C nerve fibers have been identified in hairy skin. The process of mechanotransduction requires the conversion of a mechanical stimulus into electrical signals (action potentials) through the activation of mechanosensible ion channels present both in the axon and the periaxonal cells of sensory corpuscles (i.e., Schwann-, endoneurial- and perineurial-related cells). Most of those putative ion channels belong to the degenerin/epithelial sodium channel (especially the family of acid-sensing ion channels), the transient receptor potential channel superfamilies, and the Piezo family. This review updates the current data about the occurrence and distribution of putative mechanosensitive ion channels in cutaneous mechanoreceptors including primary sensory neurons and sensory corpuscles.

## 1. Introduction

Tactile sensation is one of the most important components of mechanosensation, and originates in nerve fibers that can be distinguished based on the morphology of their skin terminals (i.e., free nerve endings and sensory corpuscles), as well as on the conduction speed of their action potentials. The sensory corpuscles are the receptors responsible for tactile modalities including light brush, touch, pressure sensation, stretch, and vibration [1,2,3]. These mechanosensitivity modalities depend on Aβ, Aδ and C nerve fibers (distinguished according to axon diameter, degree of myelination, and axonal conduction velocity) connected to low-threshold mechanoreceptors (LTMRs). 

LTMR sensory neurons are pseudo-unipolar, and the axonal processes that extend to the skin are associated with specialized cells: Merkel cells (forming Merkel cell–neurite complexes), Schwann-like cells that form part of the sensory corpuscles (Meissner corpuscles, Ruffini’s corpuscles, Pacinian corpuscles), or cells of hair follicles (sensory nerve endings associated to hair follicles) [3,4,5,6]. Aβ fibers originate in intermediate- or large-sized mechanosensory neurons and are the main fiber type mediating discriminative touch [4,7,8], although Aδ fibers [9] and C fibers [10] from small-sized neurons are also involved in mechanosensation.

Mechanotransduction is defined as the conversion of mechanical stimuli into electrical signals, and this process occurs at the periphery of LTMRs, inside the sensory corpuscles [11,12,13]; in this context, the sense of touch is a prime example of mechanotransduction in biology [14,15,16]. Each morphotype of sensory corpuscle is assumed to detect different qualities of touch. Therefore, understanding mechanotransduction in free nerve endings and sensory corpuscles requires the identification of the various molecular mechanisms that translate cell-tissue deformation into action potential firing in the corresponding LTMR.

Classically, both the mechanical properties of periaxional cells of the sensory corpuscles and differentiations on the axonal membrane were considered necessary and sufficient to generate the so-called receptor potential and consequently the action potential (see for review [3,6,17]). Nevertheless, the discovery that certain ion channels are at the basis of sensitivity, and that mechanical forces can trigger some ion channels (mechanosensitive or mechanically gated ion channels) support that mechanotransduction occurs via those ion channels. Consistently, it is believed that LTMRs innervating the skin [18,19,20,21,22,23,24] and their cutaneous target cells [25] display ion channels activated by force or displacement. In other words, gating of ion channels present in cutaneous sensory corpuscles in response to mechanical stimuli is the first step to transduce mechanical energy into electrical activity [18,20,23,26].

The present review is a compilation of the current knowledge regarding the occurrence of putative mechanosensitive ion channels in sensory corpuscles that are functionally mechanoreceptors. It was focused on ion channels belonging to the degenerin/epithelial sodium, acid-sensing, transient receptor potential, mechanosensitive potassium, and Piezo families. 

## 2. Cutaneous Mechanoreceptors

The body surface of mammals is covered by two structural types of skin, i.e., non-hairy, or glabrous, and hairy skin. Glabrous skin contains no hairs, has a thick epidermal layer, and is restricted to zones characterized by high discriminative touch (shape, size, texture) as the palm of hands or the plant of foot. Hairy skin covers more than 90% of the body surface, has a thin epidermal layer and is strongly associated with affective touch [3].

### 2.1. Glabrous Skin

**Figure 1:**
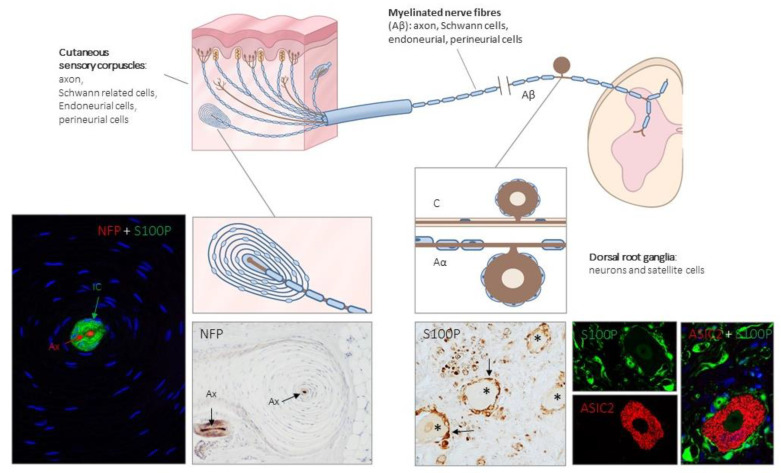
Schematic representation of the afferent innervation of mammalian glabrous skin. Glabrous skin is supplied by myelinated and non-myelinated nerve fibers (Aβ, Aδ, C), originated from large, intermediate and small sized neurons (low-threshold mechanoreceptors (LTMRs) and nociceptors) localized in the dorsal root ganglia (DRG). Aβ nerve fibers end in the dermis forming different morphotypes of sensory corpuscles. Photos on the left side correspond to sections of Pacini’s corpuscles immunostained for neurofilament proteins (NFP) and S100 protein (S100P) to, respectively, label the axon (Ax; red immunofluorescence) and the Schwann-related cells (IC: inner core; green fluorescence). Right side photos correspond to a section of human lumbar DRG—immunostained for S100P and acid-sensing ion channel protein 2 (ASIC2). S100 protein labels satellite cells (arrows and green fluorescence) while neuronal cell bodies (asterisks and red fluorescence) display ASIC2 positivity.

**Figure 2:**
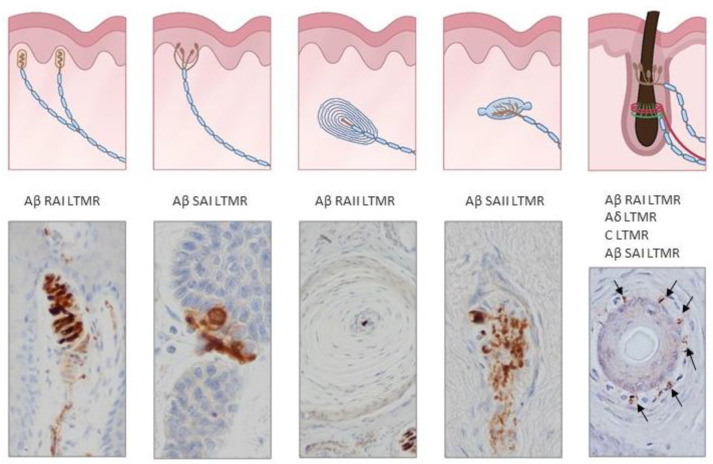
Schematic representations and photos of the different sensory corpuscle morphotypes present in human digital glabrous and hairy skin. Aβ LTMRs contact with epithelial Merkel cells or Schwann-like cells forming Merkel cell–neurite complexes (Aβ slowly adapting (SA)I-LTMRs), Meissner corpuscles (Aβ rapidly adapting (RA)1-LTMRs), Pacinian corpuscles (Aβ RAII-LTMRs) and Ruffini endings (Aβ SAII-LTMRs). Hairs have a complex nervous apparatus that consists of lanceolate and circumferential endings as well as free nerve endings; occasionally, hairs have associated Merkel cells, Ruffini and even Pacinian corpuscles. Photos were obtained from sections of human digital and facial skin immunostained for neuron-specific enolase to label the central axon, i.e., the ending of Aβ low-threshold mechanoreceptors.

In the glabrous skin, four types of terminals associated with LTMRs have been functionally identified, each associated with different cells or sensory corpuscles, all of which have more or less specific tuning properties: Merkel cell–neurite complexes, Ruffini corpuscles, Meissner’s corpuscles and Pacinian corpuscles [2,3,4,5,6,27] (Figure 1 and Figure 2).

Structurally, cutaneous sensory corpuscles consist of a central axon, surrounded by non-myelinating Schwann-like cells variably arranged, and a capsule of endoneurial and/or perineurial derivation [6,17,28,29]. It must be emphasized that although the term central axon is widely used to denominate the neuronal component of sensory corpuscles, it actually represents the peripheral process of a pseudo-unipolar neuron, localized in dorsal root ganglia (DRG) or the sensory ganglia of the cranial nerves. Therefore, the so-called central axon corresponds sensu stricto to a dendrite, or better a dendritic zone as denominated classically (see [6,30]). Filling the spaces among the cells there is a chemically complex extracellular matrix [31,32,33].

Cutaneous sensory corpuscles represent differentiated morphotypes of the Aβ LTMRs end organs. They fall functionally into two main categories: rapidly adapting (RA) and slowly adapting (SA) mechanoreceptors, which each can be sub-divided into two variants, type I and type II [3,27]. SAI mechanoreceptors are associated with epidermal Merkel cell–neurite complexes and are tuned by both static and dynamic stimuli. SAII mechanoreceptors are thought to be located in dermal Ruffini’s corpuscles although other sensory corpuscles are presumed to function as SAII [34] and are particularly sensitive to stretch. RAI and RAII mechanoreceptors are Meissner’s and Pacinian sensory corpuscles, respectively; Meissner’s corpuscles detect movement across the skin, and Pacinian corpuscles respond to vibrations [3,27].

#### 2.1.1. Merkel Cell–Axon Complexes

Merkel cells are specialized epidermal cells [35] functionally connected to Aβ SAI-LTMRs to accomplish tactile discrimination of shapes and textures [36,37]. They are present in glabrous skin especially in touch-sensitive areas, such as fingertips and lips, as well as in specialized spots in hairy skin called touch domes [38,39].

The Merkel cell–neurite complex consists of two distinct but closely associated cell types: Aβ sensory neurons and the specialized epithelial cells denominated Merkel cells. The contacts between epithelial Merkel cells and the afferent terminals are synaptic-like ones (see [37]) that use glutamate [40,41], adrenalin [42], or serotonin [43] as a neurotransmitter. Recently we also found indirect evidence for an ATP-mediated neurotransmission (L. Cárcaba et al., unpublished). They also express ion channels directly related to or required for mechanotransduction (see below).

#### 2.1.2. Meissner’s Corpuscles

Meissner corpuscles are Aβ RAI-LTMRs sensitive to dynamic skin deformation, but that resolve spatial detail poorly [27,34]. They are specific to human and primate glabrous skin, and are located within the dermal papillae, concentrated in areas particularly sensitive to light touch like fingertips, palms, soles, lips, face and the skin of male and female genitalia. Meissner’s corpuscles’ size and morphology are varied, but often they present an ellipsoid morphology being 80–150 μm in length and 20–40 μm in diameter [5,6]. They consist of an axon from an Aβ nerve fiber, non-myelinating lamellar Schwann-related cells, and a more or less developed capsule of endoneurial origin [29,44].

#### 2.1.3. Pacini Corpuscles

Cutaneous Pacinian corpuscles are structurally complex specialized sensory formations localized in hypodermis, that work as Aβ RAII-LTMRs connected to Aβ sensory nerve fibers [12,13]. They are oblong shaped, usually about 1 mm in length and display a typical onion-like structure. They consist of a central axon, sheated by non-myelinating Schwann-like cells forming the so-called inner core (with proper specific arrangements at the corpuscular terminal and ultraterminal segments) [30], both surrounded by the so-called intermediate layer of endoneurial cells, and all covered by the outer core–capsule complex of perineurial cells arranged in a multilayered concentric fashion [6,28,45].

#### 2.1.4. Ruffini’s Corpuscles

Little information is available about cutaneous Ruffini’s corpuscles [4,5]. SAII-LTMRs have been extensively characterised physiologically [46,47] but not morphologically. In many cases, SAII-LTMR responses have been recorded in nerve fibres innervating a tissue [46], but no evidence of Ruffini corpuscles in such tissues was morphologically present [48,49,50]. Cutaneous Ruffini’s corpuscles are fusiform structures with tapered ends. They consist of a single axon with numerous terminal branches embedded in a core of Schwann-related cells and collagen, all surrounded by a multilayered capsule of perineurial origin [4,5,13]. Functionally they mediate stretching information [27,34].

### 2.2. Hairy Skin: The Pilo–Neural Complexes

In the hairy skin of mammals, three major types of hairs are found: guard hairs, awl/auchene hairs, and zigzag hairs, which are densely innervated by functionally distinct sensory nerve fibers: Aβ innervates guard hairs; Aδ- (or D-hair receptors) and C-fibers, respectively, innervate awl/auchene and zigzag hairs. The peripheral endings of those nerve fibers are arranged as palisades (lanceolate endings), or as collars or rings (circumferential endings) [3]. In addition, Merkel cells and rarely Pacinian corpuscles are also present in the hairy skin associated to the follicles. Aβ SAI-LTMRs and Merkel cells form complexes to detect skin indentation denominated touch domes [51]. 

**Figure 3:**
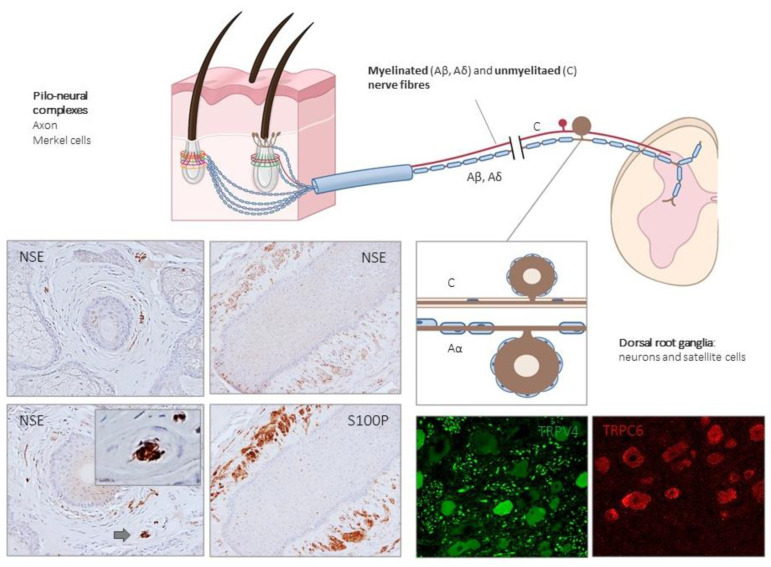
Schematic representation of the afferent innervation of mammalian hairy skin. Hairs form pilo–neural complexes with Aβ, Aδ, and C nerve fibers originated from large, intermediate and small sized primary sensory neurons localized in dorsal root ganglia (DRG). They form circumferential and longitudinal lanceolate endings around the hair follicle that work as RA-LTMRs, Aδ-LTMRs, and C-LTMRs. Guard hairs are innervated by Aβ RA-LTMR lanceolate endings; awl/auchene hairs by Aβ RA-LTMRs, Aδ-LTMRs and C-LTMRs lanceolate endings; and zigzag hairs by Aδ-LTMRs and C-LTMRs. Merkel cell touch domes are innervated by Aβ SAI-LTMRs. NSE: neuron-specific enolase; S100P: S100 protein; TRPC6: transient receptor potential canonical channel 6; TRPV4: transient-receptor potential vanilloid channel 4.

The hair follicle shaft is innervated by lanceolate and circumferential endings belonging to Aβ RA-LTMRs, Aδ-LTMRs, and C-LTMRs; lanceolate endings are mainly sensitive to movement and low-frequency vibration [52,53]. The neck of hair follicles contains unmyelinated free nerve ending LTMRs (Figure 2 and Figure 3). In mammals other than humans, D-hair receptors are the most sensitive mechanoreceptor of hairy skin, and there is practically no evidence for their existence in human hairy skin [54].

A population of unmyelinated LTMRs axons, so-called C-LTMRs, also innervate the hairy skin. The existence of C-LTMRs has been known for many decades, but is currently ignored, although they are relatively common in human skin [55]. The function of C-LTMRs is still unknown, and has been related to pleasant sensations, often associated with touch [56], and could play a role in mechanical hypersensitivity after nerve or tissue injury [57]. 

Thus, the distinct sensory functions of glabrous and hairy skin are not only defined by their neurophysiological aspects, but also have noticeable morphological differences. 

## 3. Putative Mechanosensitive Ion Channels

As mentioned previously, for many years, the genesis of the receptor potential in mechanoreceptors was explained by the mechanical properties of the periaxonal cells and the characteristics of the axon membrane forming the sensory corpuscles. Then, the discovery that mechanical forces can gate some ion channels led to the thinking that the biological basis of the sense of touch weas solved. However, almost 30 years later, the origin of the mechanotransduction remains unsolved. 

It is necessary clarify that this review is focused on the putative mechano-gated ion channels alone, since also some voltage-gated [58,59] or ligand-gated [60] ion channels are involved in mechanosensitivity; the only requirement to make them mechanosensitive is to change between ‘‘open’’ and ‘‘closed’’ states [61]. For instance, the voltage-dependent K+ channel KCNQ4 (Kv7.4) is crucial for setting the velocity and frequency preference of a subpopulation of rapidly adapting mechanoreceptors in both mice and humans [53]. Moreover, voltage-sensitive Na^+^-channels are present in the neurite and axolemma, the inner and outer lamellae in Pacinian corpuscles suggesting they are involved in both transduction and action potential generation [62]. In addition, the δ-opioid receptor (DOR) for opioids, regulates cutaneous mechanosensation, including touch, and is expressed by mechanoreceptors that form Meissner corpuscles, Merkel cell–neurite complexes, and circumferential hair follicle endings [63].

According to Delmas and Coste [26], the mechanosensitive ion channels can be divided into two categories: those responding to membrane tension and those that are susceptible to stretch. Experimental evidence suggests at least three mechanisms capable of activating the mechanically-gated ion channels: (1) modifications of cell membrane in the close vicinity of the channels; (2) tension of extracellular matrix and/or cytoskeletal proteins anchored to the extra- or intra-cytoplasmic domains, respectively, of membrane ion channels; (3) coupling of secondary mechanosensory proteins to the ion channels [18,64,65]. Therefore, any of these three mechanisms, or a combination of them, are at the basis of mechanosensitive ion channel opening and, consequently, of mechanosensing and/or mechanotransduction in sensory corpuscles. In this context, integrins [66,67] and other linking extracellular matrix proteins present in sensory corpuscles could be involved in mechanosensing and/or mechanotransduction. 

On the other hand, the relationships among the membrane, cytoskeleton and extracellular matrix are complicated because the adhesions among them are non-uniformly distributed. Furthermore, cellular membranes themselves contain spatial domains based on lipids (lipid rafts) or heterogeneous (protein corrals) and the incidence of mechanical forces inside any domain is different from that in the surrounding membrane. So, the mechanosensitive ion channels can be modulated by inclusion or exclusion from a domain (for a review see [68]).

At present, several members of the degenerin/epithelial sodium (DEG/ENa^+^C), transient receptor potential (TRP), two-pore domain potassium (K_2p_), and Piezo families of ion channels have proved to be mechanosensory and/or mechanotransducer ion channels totally or in part [1,18,20]. However, sensory phenotypes of mice deficient for these proteins do not always support a key role in mechanotransduction, and only Piezo2 has proved its mechanotransducer properties in vertebrates [23,26]. Thus, those putative mechanoproteins could be accessory proteins and not a crucial part of the proper mechanosensitive ion channel or channels.

Acid-sensing ion channels (ASICs) are a group of H^+^-gated voltage-insensitive, amiloride-sensitive cation channels included into the superfamily of degenerin/epithelial sodium channel (DEG/ENa^+^C) ion channels. Seven ASIC proteins, encoded by four genes, have been identified (ASIC1a, ASIC1b, ASIC2a, ASIC2b, ASIC3, ASIC4 and ASIC5) [69,70], and some of them are suspected to function as mechanosensors or at least be required for mechanosensation [1,18,20,71,72,73] (Table 1). Nevertheless, the role of ASIC in mechanosensitivity is doubted [74,75], although different studies have demonstrated that different isoforms of ASICs could participate in modulating it, especially ASIC2. In this way, ASIC2 knockout mice exhibit a decreased sensitivity of rapidly adapting cutaneous LTMRs and disruption of ASIC3 reduces responses of cutaneous high-threshold mechanoreceptors to noxious stimuli [76].

Transient receptor potential (TRP) ion channels are a superfamily of structurally homologous cation channels formed by seven families that include at least 28 different TRP proteins [77,78]. Nearly all TRP families have potential mechanosensory members [79,80] (Table 1). However, it has not been fully determined whether these ion channels are mechanosensors or are only required for mechanosensation [81]. Recently, it was demonstrated that mammalian members of different families of TRP channels are insensitive to membrane stretch, suggesting that they do not represent the primary mechanotransducers [82]. In spite of this, evidence is accumulating that members of TRP families participate in mechanosensing. TRPA1, belonging to the ankyrin family, could have a potential role in mediating mechanically activated currents [83] and may play a modulatory role in noxious mechanosensitivity in a subpopulation of dorsal root ganglia neurons [84,85]. Members of the canonical subfamily of TRP channels (TRPC) also participate in touch. The TRPC1, TRPC3, TRPC5 and TRPC6 channels may have a combinatorial role in mediating specific sensory functions [86]. Furthermore, the TRPC1 channel alone has a role in mechanotransduction since TRPC1-deficient mice showed a decreased by nearly 50% in slowly adapting Aβ-fibers innervating Merkel cells [87]. Furthermore, TRPV2 and TRPV4, belonging to the vanilloid TRP family, are candidates to be mechanosensors. TRPV4 plays a minor role in mechanotransduction processes in vivo [88,89], and TRPV2 has a critical role in mechanical nociception in the adult somatosensory system [90].

The family of *mechanosensitive potassium channels* regulate the activity of mechanosensitive cation channels. They fall into three classes: voltage gated (Kv) and calcium activated (Kca), inwardly rectifying channels (Kir) and “two pore domain” channels (K2P) [91,92]. K2P channels are mammalian mechanically activated ion channels, that comprise six subfamilies of channels codified by 15 distinct genes [93,94,95,96,97] and three channels in particular, TREK-1, TREK-2 and TRAAK, are the major channels showing direct mechanical gating by membrane stretch [98,99,100,101]. 

More recently the proteins codified by the *Piezo* gene, Piezo1 and Piezo2, have proved their true mechanosensory ability, and thus their direct involvement in mechanotransduction. Piezo channels are composed of an evolutionary conserved family of proteins, with a molecular divergence between Piezo1 and Piezo2 proteins beginning in vertebrates. Both Piezo1 and Piezo2 are nonselective cation channels with ~42% identity and the structure and mechanogating properties of both Piezo1 and Piezo2 have been elucidated recently [102,103,104]. They function as mechanotransducers in several somatic cells [24,105,106,107,108,109], while only Piezo2 functions as a transducer in LTMRs (see below).

ENa^+^C/Degenerin: Epithelial Na^+^ channels/degenerin; β and γ subunits of ENa^+^C; ASIC: acid-sensing ion channels; TRP: transient receptor potential ion channels—A: ankyrin family, C: canonical family, V: vanilloid family.

## 4. Putative Mechanoproteins in Mechanoreceptors

Cutaneous sensory corpuscles and Merkel cell–neurite complexes, as well as some free nerve endings, express mechanically-gated ion channels responsible for originating electric activity after a mechanical stimulus, which is sent to the central nervous system for processing and interpreting, resulting finally in a touch sensation. Zimmerman and co-workers [3] say

*“Like individual instruments in an orchestra, each LTMR subtype conveys a specific feature of the forces acting on the skin, collectively culminating in a musical symphony of neural impulses that the brain translates as a touch”.*

**Figure 4:**
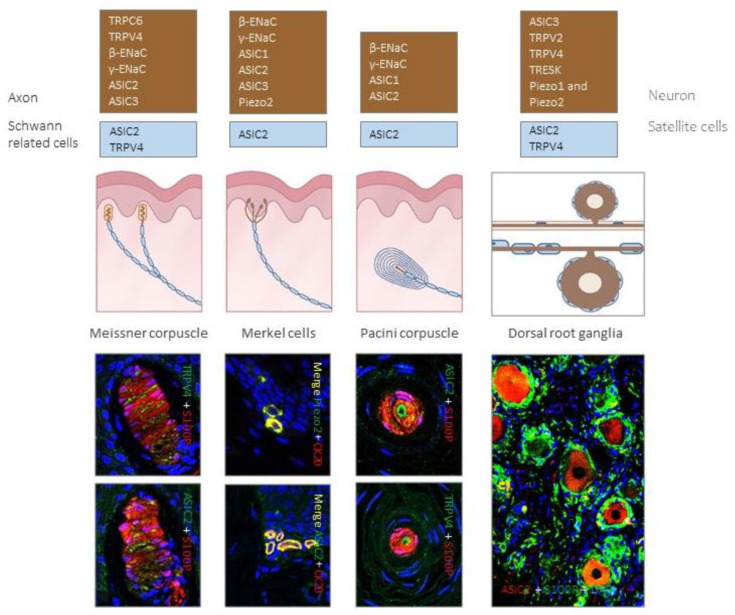
Expression of different putative mechanoproteins in cutaneous sensory corpuscles and human lumbar dorsal root ganglia (DRG). Brown boxes contain ion channels identified in LTMR axons or cells bodies, while blue boxes contain ion channels localized in Schwann-related cells of sensory corpuscles and satellite cells of DRG. Photos show localization of putative mechanoproteins in human cutaneous sensory corpuscles, Merkel cells and lumbar DRG.

Although this review is focused on the periphery of the nervous system, the occurrence of putative mechanoproteins in cutaneous mechanoreceptors has been mostly studied in parallel with LTMR neurons (see [21,105,109]). For this reason, a reference to DRG neurons will be included (Figure 4). Here we detail the localization of putative mechanoproteins in mammalian mechanoreceptors, with special reference to humans. 

### 4.1. Degenerin-ENa^+^C/ASIC Channels

Both subunits β-ENa^+^C and γ-ENa^+^C, but not α-ENa^+^C, have been detected in Merkel cell–neurite complexes, Meissner-like corpuscles, and the axon of murine Pacinian corpuscles [111,112]. Consistently, β-ENaC and γ-ENaC were detected in in murine DRG [111]. 

The presence of ASICs in cutaneous mechanoreceptors has been well studied in rodents, primates and humans by immunohistochemistry [110]. In mice, ASIC2 and ASIC3 were localized in the axons supplying Meissner-like and Pacinian corpuscles, as well as in Merkel disks [111,112]. moreover, ASIC2 immunoreactivity was detected in the inner core of Pacinian corpuscles [112]. In hairy skin, ASIC2 and ASIC3 were present in the palisades of lanceolate nerve endings and in free myelinated nerve endings [76,113,114]. Immunoreactivity for ASIC2 was also found in axons supplying Meissner and Pacinian corpuscles of *Macaca fascicularis* [115].

Data in humans are scarce. Using immunohistochemistry, ASIC1 was detected in the central axon of Pacinian corpuscles whereas ASIC2 was present in the inner core, and ASIC3 was undetectable [116]. In a detailed study carried out in human digital skin and lumbar DRG, ASIC2 was found in the axons of Merkel cell–neurite complexes, Meissner and Pacinian corpuscles; moreover, a variable percentage of Meissner (8%) and Pacinian corpuscles (27%) also displayed ASIC2 Immunoreactivity in the lamellar and inner core Schwann-related cells. Almost all intermediate or large sized neurons in DRG (mean diameter ≥ 20–70 µm) were ASIC2 positive, and thus compatible with a mechanosensory neuron phenotype [117]. 

### 4.2. TRP Ion Channels

Almost all families of TRP ion channels have a member that can gate different modalities of force and movement, but until now only a few are serious candidates to be actually mechanosensitive. 

TRPC1, TRPC3, TRPC5, TRPC6 [86,87,98,118,119], TRPV4 [88,89,120], TRPV2 [90,121], and TRPA1 [18,83,84,85] seem to participate in mechanosensing.

In particular, TRPC6 is involved in light touch [118,119] and contributes to cutaneous mechanosensation in combination with TRPC3 [86]. TRPC6 is found in dorsal root ganglia neurons [122] as well as in the axon of human Meissner corpuscles, both alone or co-localized with TRPV4 [123]. Messenger RNAs for TRPV4 and TRPC6 are frequently co-expressed in sensory neurons [118]: TRPC6 is mainly present in small neurons [119,124,125] and TRPV4 in both small and large neurons [88,126,127,128]. This co-localization can be of interest, since TRPC6 cooperates with TRPV4 See comment in PubMed Commons below to produce mechanical hyperalgesia, presumably as part of a mechanoreceptor signaling complex. On the other hand, TRPC1 is also a candidate to be a mechanical sensitive channel as it is related with SAI-LTMR neurons that innervate Merkel cells [87]. 

Some TRP vanilloid channels also have mechanosensory functions [89,120,128]. In mammals other than humans, TRPV4 was detected in Meissner corpuscles, Merkel cells, penicillate nerve endings and intraepidermal terminals, but it has not been demonstrated on hair follicle palisades [126,128]. Regarding humans, TRPV4 immunoreactivity was found in Meissner corpuscles, mainly in the central axon but also occasionally in lamellar cells [123] and DRG neurons [126,127]. Mutations leading to TRPV4 deficit involve decreased responsiveness to sensation of noxious mechanical stimuli in mice [129], while TRPV mutations cause peripheral neuropathies in humans [130]. Mechanical hyperalgesia is related to TRPV4 and TRPC6 channels, probably within a mechanoreceptor related signaling complex.

### 4.3. Piezo2

Piezo2 is the first ion channel that fulfills the properties of mechanosensoy ion channels, and directly participates in mechanotrasnduction [131,132,133,134,135] by modulating the conversion of touch to itch [136]. It is expressed in 20–50% of mammalian DRG, including LTMRs and nociceptors [105,107]. In murine skin it is present in Merkel discs and isolated Merkel cells [107,131,132,133,134,135], Meissner-like corpuscles and lanceolate nerve endings [105]. 

Piezo2 has been also detected in human Merkel cells and Meissner´s corpuscles axon, in an age-dependent manner [137,138]. Recently we have also detected Piezo2 in the axon of human cutaneous Pacinian corpuscles (García-Piqueras, J. et al., unpublished). Consistently with those localizations, Piezo2-deficient mice show an almost complete deficit in light-touch sensation and proprioception with preserved function in other somatosensory modalities [105]. Piezo2 mutations in human patients lead to selective loss of touch perception and heavily decreased proprioception [139,140].

## 5. Concluding Remarks and Perspectives

LTMR neurons terminate in the skin as specialized sensory organs, and represent the peripheral tip of sensory afferents. They are closely related to non-neuronal cells such as Merkel cells and Schwann-related cells. Currently, different ion channels are assumed to participate in mechanosensation and/or mechanotransduction processes. These channels have been localized not only in LTMR endings, but also in non-neuronal cells, thus suggesting a role in mechanosensation for these cells. Non-neuronal cells were considered indirect elements for the transduction process, being traditionally limited to neurons mechanical filtering and/or trophic support. However, there is current evidence that presynaptic components and neurotransmitters are synthesized by non-neuronal cells, and probably released to LTMRs, which, in turn, express the receptors for some of these neurotransmitters. This has been demonstrated in the case of Merkel cell–neurite complexes, and Pacini corpuscles [25,37,141], but not for other mechanoreceptors. 

These findings, together with the mechanosensitive ion channel expression by Merkel cells and sensory corpuscles glial-related cells add complexity, and new enigmas appear regarding peripheral mechanosensibility and mechanotransduction mechanisms. This review is focused on a reduced number of ion channels that have demonstrated participation in mechanosensation and/or mechanotransduction. The most solid of these channels is Piezo2, an essential component in mechanotransduction. However, the molecular mechanism of both processes is only partially known. For example, a KCNQ4 potassium voltage-gated channel is required for proper velocity coding and frequency tuning in peripheral lanceolate endings of Aβ-hair follicle afferents, and in Meissner corpuscles, in both mice and humans [53]. Interestingly, D-hair lanceolate endings do not express the KCNQ4 channel [53,142] and express instead CaV3.2 calcium T-type voltage-gated [143,144] and KCNQ (Kv7) channels [145]. Can these ion channels, and probably other undiscovered ones, tune sensory corpuscles? Does the prominent extracellular matrix have a role modulating not only mechanical inputs but also the proper mechanotransducers? These and other questions remain open, and will be solved in future research regarding the mechanisms of cutaneous mechanosensitivity. 

## Figures and Tables

**Table 1:** Mechanosensitive ion channels in mammals.

**ENa^+^C/Degenerin**	β-ENa^+^C, γ-ENa^+^C ASIC1, ASIC2, ASIC3
**TRP**	TRPA1, TRPC1, TRPC3, TRPC6, TRPV2, TRPV4
**Two-Pore Domain K^+^**	TREK1, TREK2, TRAAK2
**Piezo**	Piezo1, Piezo2

Based on Del Valle et al. (2012) [110] and Gu and Gu (2014) [21].
